# FXR expression in rats of hilar cholangiocarcinoma

**DOI:** 10.1038/s41598-022-12850-w

**Published:** 2022-05-24

**Authors:** Meng-yu Zhang, Ming Luo, Jie-ping Wang

**Affiliations:** 1 Department of General Surgery (Hepatobiliary Surgery), The Affiliated Hospital of Southwest Medical University, Luzhou, 646000 China; 2 Department of Rehabilitation, The Affiliated Hospital of Southwest Medical University, Luzhou, 646000 Sichuan Province China

**Keywords:** Cancer, Cancer models

## Abstract

The study objective was to detect the expression of farnesoid X receptor (FXR) in a rat model of hilar cholangiocarcinoma to provide a new therapeutic target for gene therapy in hilar cholangiocarcinoma. Sixty male Wistar rats (weighing 190 ± 8 g) were randomly divided into three groups (experimental group, control group and sham operation group, 20 rats in each group). The three groups were fed a standard diet. The QBC939 cell suspension of cholangiocarcinoma was injected into the hilar bile duct in the experimental group with a microsyringe. The control group was injected with normal saline, and the sham operation group was not injected with any drugs. A modified tail suspension test (TST) was used to evaluate the mental state and physical activity of rats every day. At 5 weeks, one rat in the experimental group was euthanized, and the changes in the hilar bile duct were recorded. The procedure was repeated at one and half months. After one and half months, hilar cholangiocarcinoma only occurred in the experimental group. Pathological examination confirmed the formation of tumours, and hilar bile duct tissues were taken from the three groups. FXR expression in the hilar bile duct was detected by real-time polymerase chain reaction (RT–PCR) and immunohistochemistry. After two weeks, the rats in the experimental group ate less, and their weight was significantly reduced. One and half months later, hilar cholangiocarcinoma was detected in 16 rats in the experimental group. The levels of alanine aminotransferase and aspartate transaminase in the experimental group were higher than those in the other two groups. The ratio of FXR/GAPDH mRNA was significantly different among the hilar cholangiocarcinoma, control and sham operation groups. Under the light microscope, FXR protein reacted with anti-FXR antibody and showed granular expression. Every pathological section included 4800 cells. A total of 1856 positive cells were in the experimental group, 3279 positive cells were in the control group, and 3371 positive cells were in the sham operation group. FXR expression in the hilar cholangiocarcinoma of rats was significantly lower than that in normal hilar bile duct tissues, suggesting that drugs targeting FXR may be a new strategy for the treatment of hilar cholangiocarcinoma.

## Introduction

Malignant tumours may appear in all parts of the liver and biliary system, and hilar cholangiocarcinoma is one of the malignant tumours of the biliary system. Although its incidence rate is not high, its malignancy rate is very high. According to the statistics of medical institutions at all levels, the mortality of hilar cholangiocarcinoma is high. Patients with hilar cholangiocarcinoma in the early stage have no obvious symptoms because the bile duct has not been completely blocked. With the further development of the disease, when most of the biliary tract is blocked or when it is completely blocked, jaundice, impaired liver function and acute cholangitis may occur. At this time, the disease has entered the middle and late stages, and treatment is very difficult^1–3^. Surgery is the preferred method, but the number of patients who can undergo surgery is very limited. According to the Bismuth Corlett classification, only type I and type II hilar cholangiocarcinomas meet the requirements of radical resection, but type III and type IV hilar cholangiocarcinomas often cannot be completely resected. Some patients can have palliative surgery, but the 5-year survival rate is low. Some patients could not get the chance of operation because of tumour invasion, metastasis, cardiopulmonary insufficiency and severe liver function damage. Alternative therapies include immunotherapy, radionuclide therapy, chemotherapy, interventional therapy, and radiofrequency ablation. However, after clinically applied, they cannot significantly improve the survival rate. Therefore, there is an urgent need for new treatments.

Gene therapy is a new method in tumour therapy developed in recent years that has been used in the treatment of liver cancer and gastric cancer and has achieved good results. The combination of gene therapy and other treatment methods improves the survival rate of patients and the quality of life. However, few target genes have been found in hilar cholangiocarcinoma, and gene therapy has not been widely used. Therefore, researchers are trying to identify genes related to the pathogenesis of hilar cholangiocarcinoma as therapeutic targets. Farnesoid X receptor (FXR) is related to bile acid metabolism, and its gene and protein expression plays an important role in the occurrence and development of colon cancer, breast cancer and liver cancer^4–6^. Insook et al. found that mice without the FXR gene developed hepatocellular carcinoma on the basis of liver cirrhosis. In previous experiments, we found that NTCP and FXR expression levels showed an opposite trend in the hyperlipidaemic rat model. The expression level of NTCP in the hilar cholangiocarcinoma rat model was higher than that in the normal bile duct tissue because NTCP is the target gene of FXR and the inhibitor of FXR. Thus, we studied the expression of FXR in hilar cholangiocarcinoma and whether its role in hilar cholangiocarcinoma was enhanced or decreased, and we investigated whether FXR inhibitors have an effect on its expression in tumour tissues. We hope to find a new target for the treatment of hilar cholangiocarcinoma.

## Materials and methods

### Statement

The study was carried out in compliance with the ARRIVE guidelines.

### Rats

Sixty Wistar rats (male, 190 ± 8 g) were provided by the Animal Experimental Center of Southwest Medical University. They were randomly divided into three groups (the experimental group, the control group and the sham operation group, n = 20 each). Before the study, the rats were healthy and were not administered any drugs.

### Experimental methods

The materials used include the following: Microsyringes (HAMILTON, Switzerland), pentobarbital sodium (Beijing Younikang Biotechnology Co., Ltd.), the QBC939 human cholangiocarcinoma cell line (Shanghai Tongpai Biotechnology Co., Ltd.), DMEM (Sigma Inc.), anti-FXR antibody (Chemicon USA), and ABI 7500 real-time PCR detection system (USA). Frozen tissue sections were prepared using a freezing microtome, liquid nitrogen, and OCT embedding agent. The main reagents were dNTPs, buffer solution, TRIzol, chloroform, and real-time PCR kits (SR1100). The sense and antisense primers used to detect FXR mRNA levels were as follows: 5'-CCTCATTGTCTCCCCGACTT A-3' and 3'-GCCTCTAGAAAGCAGTGTTCA-5'. The sense and antisense primers used to detect *Gapdh* mRNA levels were as follows: 5′-GATGGTGGGTATGGGTCAGAA-3′ and 3′- CTAGGAGCCAGGGCAGTAATC-5′. The 2-∆∆Ct method was used to normalize the data. The diameter of the needle tip of the microsyringe was 40 μm and was connected to a 100 µL syringe through a rubber tube.

Establishment of the animal model was performed, and all Wistar rats were fed a standard diet. QBC939 human cholangiocarcinoma cells were cultured in DMEM at 37 °C and 5% saturated humidity. The cells with poor growth were filtered out, and the cells with good growth were retained. After the cells with good growth were prepared into a cell suspension with a concentration of 1 × 10^6^ cells/mL, Wistar rats were inoculated. The experimental group was anaesthetized with 1.5% pentobarbital sodium and 0.2 ml/100 g intraperitoneal injection. After disinfection, the abdomen was cut along the median line. Then, the microinjector needle was inserted into the hilar bile duct, and 100 µL of tumour cell suspension was injected. The bleeding was stopped by pressing, the abdominal layers were closed in turn, and the operation was complete. Normal saline was injected into the bile duct in the control group, and nothing was injected into the sham operation group. The mental state, diet and physical activity of the rats were evaluated daily by comprehensive behaviour scores and Basso Beattie Bresnahan assessment. At 5 weeks, one rat in the experimental group was euthanized, and the changes in the hilar bile duct were recorded. The procedure was repeated at one and half months. Pathological examination confirmed the formation of tumours, and hilar bile duct tissues were taken from the three groups. RT–PCR was used to detect the expression levels of FXR mRNA (RNA was extracted from hilar cholangiocarcinoma and normal hilar bile duct), and GAPDH expression level served as an internal control. Immunohistochemistry was used to analyse the expression of FXR protein. Under the light microscope, FXR protein reacted with anti-FXR antibody and showed granular expression. Every pathological section was randomly divided into 6 regions, and 80 cells were observed in each region. Tissues with positive cells at a rate of > 10% were considered positive, and those with positive cells at a rate of < 10% were considered negative.

### Statistical analysis

Data are presented as the mean ± SD. SPSS 22.0 statistical software was used for data analysis. The *t* test was used to judge the differences between two groups. The χ^2^ test was used to evaluate immunohistochemistry data, and *P* < 0.05 was used to indicate statistically significant differences.

### Ethics approval and informed consent

The study protocol was approved by the Ethics Committee of the affiliated hospital, Southwest Medical University, Luzhou, Sichuan Province, China. Number:2020415. Animal welfare guidelines abided by China Laboratory Animal Welfare Law and Animal management regulations, Number: GB/T 35892‐20181.

### Consent for publish

All authors have approved the manuscript and agree with publication in Scientific Reports.

## Results

**Figure 1:**
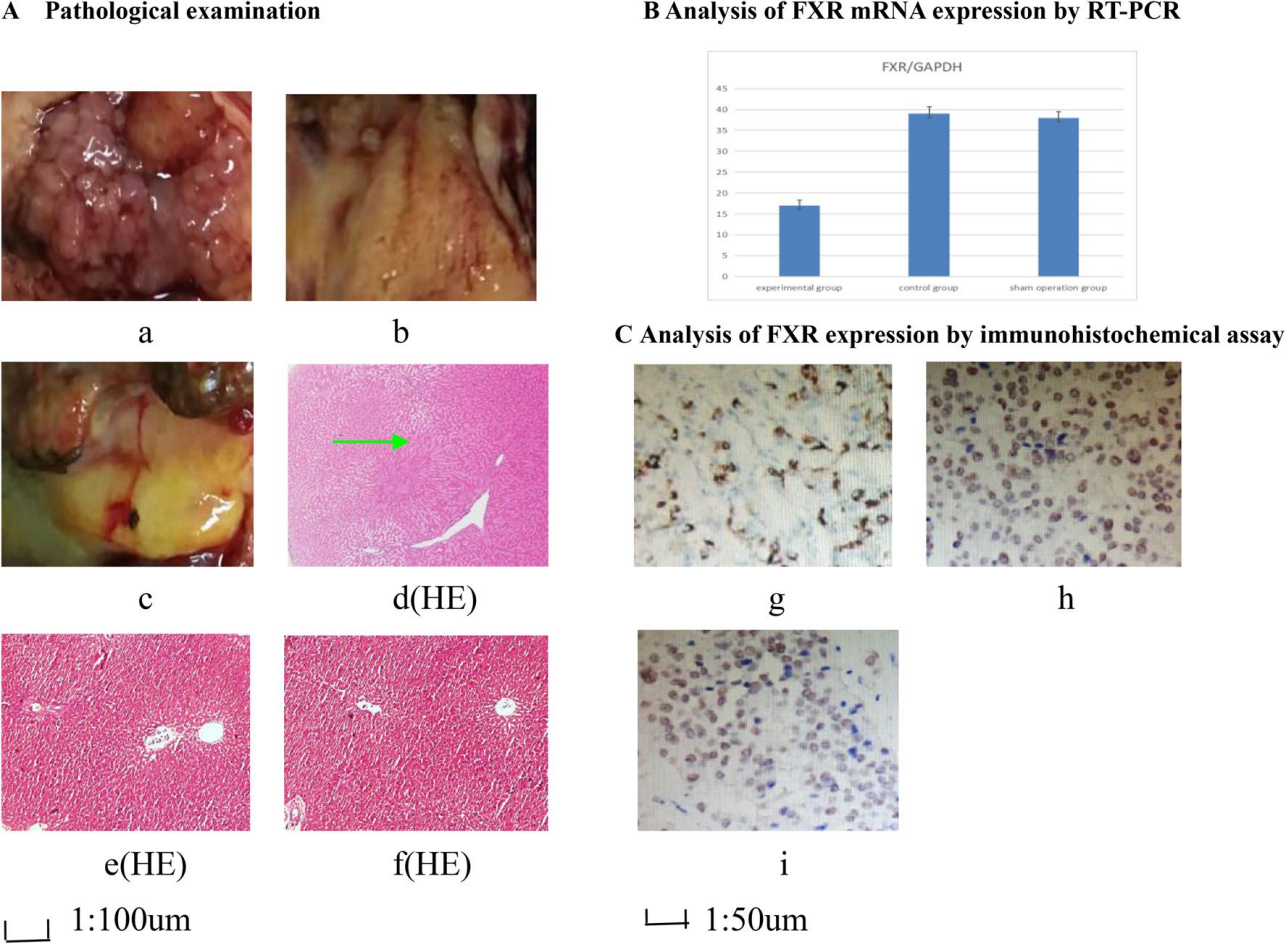
Pathological examination, RT-PCR and immunohistochemical assay. (**A**) Pathological examination. (a) hilar cholangiocarcinoma in experimental group; (b) hilar bile duct in control group. (c) hilar bile duct in sham operation group; (d) the lobulated masses are indicated by the green arrow in hilar cholangiocarcinoma. HE stain (magnification 100 ×). (e) hilar bile duct tissues in control group. HE stain (magnification 100 ×). (f) hilar bile duct tissues in sham operation group. HE stain (magnification 100×). One and half months later, hilar cholangiocarcinoma was detected in 16 rats in the experimental group, they are lobulated masses. But in control group and sham operation group only there were mild inflammation and edema in hilar bile ducts, and a small amount of inflammatory cell infiltration. The tumour cells were multinucleated and the endoplasmic reticulum was swollen. The cells in the control group and the sham operation group were slightly edematous. (**B**) Analysis of FXR mRNA expression by RT-PCR. Through RT-PCR we found that in hilar cholangiocarcinoma, control group and sham operation group the FXR/Gapdh ratios were 17 ± 1.3, 39 ± 1.6 and 38 ± 1.5, respectively. After eight cycles, there was significant statistical difference among the three groups (between experimental group and control group, t = 3.217, P < 0.05, between experimental group and sham operation group, t = 3.185, *P* < 0.05). (**C**) Analysis of FXR expression by immunohistochemical assay. (g) FXR expression in hilar cholangiocarcinoma of experimental group (magnification 200 ×). (h) FXR expression in normal hilar bile duct of control group (magnification 200×). (i) FXR expression in normal hilar bile duct of sham operation group (magnification 200×). FXR protein reacted with the anti-FXR antibody, and FXR protein expression is shown. Every pathological section included 4800 cells. 1856 positive cells (38.7%) were in the experimental group 3279 positive cells (68.3%) were in the control group, and 3371 positive cells (70.2%) were in the sham operation group, there was significant statistical difference among the three groups (χ^2^ = 33.97, *P* < 0.05, between experimental group and sham operation group. χ^2^ = 33.16, *P* < 0.05, between experimental group and control group.).

After two weeks, the rats in the experimental group ate less, and their weight was significantly lower than those of the other two groups. (Tables 1, 2). Three rats in the experimental group died after 6 weeks. There were no fatalities in the other two groups. Through pathologic examination, we detected hilar cholangiocarcinoma in 16 rats (80%) in the experimental group after six weeks (Fig. 1A). The changes in liver function in the three groups are shown in Table 3.

**Table 1:** Daily food-intake (g).

Week	Control group (n = 20)	Sham operation group (n = 20)	Experimental group (n = 20)
2	18.92 ± 0.05	19.05 ± 0.07	19.01 ± 0.06
3	23.21 ± 0.11	23.58 ± 0.12	21.85 ± 0.10
4	26.93 ± 0.08*	27.02 ± 0.09^^^	19.47 ± 0.05
5	28.89 ± 0.12^#^	29.10 ± 0.13^&^	18.14 ± 0.03

*P < 0.05 compared with the experimental group, ^^^*P* < 0.05 compared with the experimental group, ^#^*P* < 0.05 compared with the experimental group, ^&^*P* < 0.05 compared with the experimental group. After 2 weeks the rats in experimental group ate less, and their weight was significantly reduced compared with the other two groups.

**Table 2:** Body mass (g).

Week	Control group (n = 20)	Sham operation group (n = 20)	Experimental group (n = 20)
2	206 ± 3.8	207 ± 4.1	207 ± 4.3
3	220 ± 4.5	221 ± 4.9	220 ± 4.7
4	237 ± 5.8*	238 ± 6.1^^^	205 ± 4.2
5	250 ± 6.2^#^	251 ± 6.4^&^	196 ± 3.9

*P < 0.05 compared with the experimental group, ^^^*P* < 0.05 compared with the experimental group, ^#^*P* < 0.05 compared with the experimental group, ^&^*P* < 0.05 compared with the experimental group. After 2 weeks the rats in experimental group ate less, and their weight was significantly reduced compared with the other two groups.

**Table 3:** Changes of liver function.

Related indicators	Control group (n = 20)	Sham operation group (n = 20)	Experimental group (n = 20)
(ALT)/(U/L)	72.97 ± 2.53^$^	73.05 ± 2.59^$$^	155.32 ± 4.36
(AST)/(U/L)	78.51 ± 2.67^&^	79.26 ± 2.74^&&^	160.28 ± 4.82
(TC)/(mmol/L)	2.46 ± 0.11*	2.50 ± 0.13**	6.52 ± 0.15
(TBA)/(μmol/L)	1.67 ± 0.05^#^	1.69 ± 0.06^##^	5.08 ± 0.11
(TBIL)/(μmol/L)	3.85 ± 0.04	3.87 ± 0.06	3.86 ± 0.04
(DBIL)/(μmol/L)	0.71 ± 0.03^^^	0.73 ± 0.05^^^^	2.13 ± 0.09

^$^*P* < 0.05 compared with the experimental group, ^&^*P* < 0.05 compared with the experimental group, *P < 0.05 compared with the experimental group, ^#^*P* < 0.05 compared with the experimental group, ^^^*P* < 0.05 compared with the experimental group. ^$$^*P* < 0.05 compared with the experimental group, ^&&^*P* < 0.05 compared with the experimental group,**P < 0.05 compared with the experimental group, ^##^*P* < 0.05 compared with the experimental group, ^^^^*P* < 0.05 compared with the experimental group. The levels of total cholesterol, total bilirubin, direct bilirubin, alanine aminotransfease and aspartate transaminase, in experimental group were higher compared with the other two groups. ALT: alanine aminotransfease, AST: aspartate transaminase, TC: total cholesterol, TBA: total bile acids, TBIL: Total bilirubin, DBIL: direct bilirubin.

### Analysis of FXR expression

Through RT–PCR, we found that in the hilar cholangiocarcinoma, control and sham operation groups, the *FXR*/*Gapdh* ratios were 17 ± 1.3, 39 ± 1.6 and 38 ± 1.5, respectively. After eight cycles, there was a significant difference among the three groups (between the experimental group and the control group, *t* = 3.217, *P* < 0.05; between the experimental group and the sham operation group, *t* = 3.185, *P* < 0.05) (Fig. 1B).

*FXR* protein reacted with the anti-*FXR* antibody, and *FXR* protein expression is shown in Fig. 1C. Every pathological section included 4800 cells. A total of 1856 positive cells (38.7%) were in the experimental group, 3279 positive cells (68.3%) were in the control group, 3371 positive cells (70.2%) were in the sham operation group, and there was significant difference among the three groups (χ^2^ = 33.97, *P* < 0.05, between experimental group and sham operation group; χ^2^ = 33.16, *P* < 0.05, between experimental group and control group.).

## Discussion

Among the malignant tumours of the digestive system, the degree of malignancy of biliary system tumours is high, especially for hilar cholangiocarcinoma, which is difficult to resect, and conventional treatment is difficult to effectively administer. Although in the past few years, we have known that the factors related to hilar cholangiocarcinoma include bile duct stones, primary sclerosing cholangitis and congenital cholangiectasis and that diagnostic and treatment methods have been developed in basic and clinical research, the pathophysiological changes of hilar cholangiocarcinoma, the mechanism of tumour metastasis and invasion, the changes in tumour-related gene expression and the regulatory mechanism of specific signal transduction pathways related to hilar cholangiocarcinoma are not completely clear^7–10^. For example, hilar cholangiocarcinoma may be caused by the accumulation and interaction of specific factors, but these factors that can lead to the occurrence of tumours and promote the progression of tumours may regulate each other, and it is uncertain how this is achieved, which may explain the lack of effective treatment. Usually, early or smaller tumours can be completely removed, and the prognosis is good. However, if the tumour infiltrates surrounding tissues or metastasizes, surgery is not possible. The effects of chemotherapy, microwave ablation and radiotherapy are limited. The recurrence and metastasis rates are high, and the 5-year survival rate is low. Therefore, there is an urgent need for more effective diagnostics and treatment methods. Gene therapy is a new method developed in recent years and has great potential; although it has been used in the treatment of gastric cancer and liver cancer in the digestive system, it is not widely used in the treatment of other tumours. Therefore, researchers are trying to identify genes related to the pathogenesis of hilar cholangiocarcinoma as therapeutic targets^11–13^.

FXR is usually expressed on the surface of hepatocytes and bile ducts, and its function is to regulate the secretion and reabsorption of bile acids. It is an important part of the enterohepatic circulation of bile acids. Bile acids are first synthesized by hepatocytes, and bile is transported to the intestine by a bile salt export pump (BSEP). After bile acids enter the small intestine, approximately 95% of conjugated bile acids are reabsorbed. NTCP mediates approximately 80% of the transport of bile acids into hepatocytes, and these bile acids are secreted into bile again to promote the enterohepatic circulation of bile acids^14–17^. Because BSEP and NTCP are the target genes of FXR, FXR plays an important role in stabilizing bile acid concentration. If the expression level of FXR is abnormal, disorders in bile acid secretion and reabsorption may occur. When FXR levels decrease, BSEP levels decrease synchronously, while NTCP levels increase. Therefore, bile acid secretion is insufficient, and reabsorption is excessive. The enterohepatic circulation of bile acid cannot proceed smoothly, bile will accumulate in the bile duct, and components in bile may deposit in the bile duct, leading to cell degeneration, cholangitis and stone formation^18–20^. The long-term existence of cholangitis and bile duct stones is one of the possible reasons for the development of hilar cholangiocarcinoma. Therefore, a better understanding of the expression of FXR in hilar cholangiocarcinoma plays an important role in identifying new treatment methods^4,21–23^.

Previous studies have shown that the expression level of FXR in hepatocellular carcinoma was significantly decreased, and the incidence of liver tumours in FXR gene knockout mice was 100%. FXR deletion can activate the Wnt/β-catenin and RalA-GTP carcinogenic pathways, leading to hepatocarcinogenesis. In patients with hepatitis B, FXR deletion can promote the occurrence of liver cancer by reducing the transcriptional activity of FXR-KNG1 signalling. In vitro cell experiments have demonstrated that FXR is expressed in hepatocellular carcinoma cells and that FXR agonists can inhibit the proliferation of hepatoma cells. Animal experiments have also found that FXR expression levels are increased in liver cancer tissues and that FXR agonists can inhibit tumour growth. We previously found that NTCP was expressed in liver cancer and cholangiocarcinoma in rats and its expression levels were increased in cholangiocarcinoma. Because tumours of the liver and biliary system often affect each other, we tried to analyse whether the expression of FXR, the upstream regulatory gene of NTCP, showed a similar change in the tumours of the biliary system of rats. It is uncertain whether FXR was expressed in hilar cholangiocarcinoma, whether its levels are increased or decreased, and whether FXR agonists can play a therapeutic role. Hilar cholangiocarcinoma is often difficult to radically resect in the biliary system. Therefore, we designed this experiment to preliminarily investigate the expression of FXR in hilar cholangiocarcinoma. In the study, six weeks after drug injection, an experimental model of hilar cholangiocarcinoma induced by QBC939 injection was successfully established in the experimental group. Due to the existence of tumours, the bile secretion of rats was blocked, and digestive function was seriously affected. Therefore, from the second week to the fifth week, the food intake of rats in the experimental group decreased, and then the body weight decreased. However, because there was no tumour effect, the opposite change was observed in the control group and sham operation group. Similarly, we found that the damage to liver function was more serious in the experimental group, and the levels of total cholesterol, total bilirubin, direct bilirubin, alanine aminotransferase and aspartate aminotransferase in the experimental group were higher than those in the other two groups. In other words, the bile in the experimental group had been silted after bile duct obstruction, which was an important reason for the formation of cholangitis. Simultaneously, muddy stones emerged from the bile ducts of rats in the experimental group. Through RT–PCR and immunohistochemistry, we found that the expression level of FXR in hilar cholangiocarcinoma was lower than that in the control group and sham operation group, and the differences in expression produced significant differences. Therefore, we confirmed that if the amount of bile acids in the bile duct increases, the expression of FXR level will increase^24–26^, which will accelerate the secretion of bile acids and reduce the reabsorption of bile acids to maintain the stability of enterohepatic circulation. However, in hilar cholangiocarcinoma, the expression level of FXR greatly decreased, and bile acid secretion significantly decreased. Therefore, cholestasis in the bile duct may occur, and the components in the bile may deposit to form stones, causing cell degeneration and inflammation around the bile duct, resulting in the phenomenon of bile duct cell destruction, proliferation and destruction alternately. The repeated destruction and proliferation of bile duct cells increase the possibility of cells with malignant phenotypes.

At present, there are very few drugs that can play a role in the treatment of hilar cholangiocarcinoma, and different problems have been found in the treatment process. For example, the scope of their use is very limited. On the one hand, only a small number of tumour tissues with clear pathological classification will respond to drugs. On the other hand, a large dose is needed, which has adverse effects on liver and kidney function and the digestive system, causing liver and kidney dysfunction and gastrointestinal bleeding, so tolerance to these drugs is poor. The drugs in the research and development stage do not directly target the genes related to hilar cholangiocarcinoma. Therefore, after FXR expression detection, we performed another experiment. In preliminary studies, we found that the FXR agonist obeticholic acid inhibits hilar CCA formation in rats (unpublished findings); however, these studies are still ongoing. GS-9674 and CS0159 have been proven to be powerful FXR agonists that can act on primary sclerosing cholangitis (PSC), and PSC has been proven to be one of the causes of hilar cholangiocarcinoma. Clinical trials for all these drugs have begun, and these agents may play a role in the treatment of hilar cholangiocarcinoma. These existing research results may enhance our understanding of the molecular basis of hilar cholangiocarcinoma and help us identify a new method for hilar cholangiocarcinoma treatment, but there are limitations at present. Although we found changes in FXR expression levels in hilar cholangiocarcinoma, and the information obtained in the current study can strengthen our understanding of the signalling pathway changes in hilar cholangiocarcinoma, the changes in genes related to FXR need to be further clarified. The effect and factors influencing the impact of drugs on FXR need to be further studied. In the next 5 years, there will be more research in this field to assess new treatments. From the existing experimental results, gene therapy has good development prospects in this field^27–29^. This study may help us identify new effective therapeutic targets based on the changes in FXR.

## Data Availability

All data generated or analysed during this study are included in this published article.
